# 

**Published:** 1931

**Authors:** 


### The Medical Library of the University of Bristol,

WITH WHICH IS INCORPORATED

**The Library of the Bristol Medico-Chirurgical Society.**

---

*The following donations have been received since the publication of the list in February,* 1931.

June, 1931.

| | |
|---|---|
| Messrs. Allen & Hanbury (1) .. .. | 1 volume. |
| American Laryngological Association (2) .. | 1 ,, |
| American Laryngological, Rhinological and Otological Society (3) .. .. .. | 1 ,, |
| Dr. W. B. Crow (4) .. .. .. .. | 1 ,, |
| Dental Board of the United Kingdom (5) .. | 1 ,, |
| Professor E. W. Hey Groves (6) .. .. | 5 volumes. |
| Henry Phipps Institute (7) .. .. .. | 1 volume. |
| Michell Clarke Memorial Fund (8) .. .. | 2 volumes. |
| Otago University Medical School (9) .. | 1 volume. |
| Surgeon-General of the U.S. Army (10) .. | 1 ,, |

Unbound periodicals have also been received from Professor E. W. Hey Groves.

### THE ONE HUNDRED AND THIRTY-SIXTH LIST OF BOOKS.

The figures in round brackets refer to the figures after the names of the donors. The books to which no such figures are attached have either been bought from the Library Fund or received through the *Journal.*

| | | | |
|---|---|---|---|
| **Birkett, G. E.** .. .. | *Radium Therapy* .. .. .. .. .. .. .. | | 1931 |
| **Borghi, M.** .. .. | *Il Comportamento Istologico dei Trapianti Ossei Autoplastici Viventi in Sede Ossea Chiusa* | | 1930 |
| **Brash, J. C.** .. .. | *The Ætiology of Irregularity and Mal-occlusion of the Teeth* .. .. .. .. .. .. .. .. | (5) | 1931 |
| **Corner, G. W.** .. .. | *Anatomy* .. .. .. .. .. .. .. .. .. .. .. | | 1930 |
| **Cripps, E. C.** .. .. | *Plough Court* .. .. .. .. .. .. .. .. | (1) | 1931 |
| **Crow, W. B.** .. .. | *Principles of Morphology* .. .. .. .. | (4) | 1929 |
| **Davies, T. A.** .. .. | *Primary Syphilis in the Female* .. .. .. | | 1931 |
| **Dawson, B.** .. .. | *History of Medicine* .. .. .. .. .. .. | | 1931 |
| **Dawson, W. R.** .. | *The Beginnings—Egypt and Assyria* .. .. | | 1930 |

| | | |
|---|---|---|
| **Floyer, Sir J.** .. .. | *Inquiry into the Uses and Abuses of the Hot, Cold and Temperate Baths in England* (8) | 1697 |
| **Fulton, J. F.** .. .. | *Physiology* .. .. .. .. .. .. .. .. .. .. .. | 1931 |
| **Iredell, C. E.** .. .. | *Colour and Cancer* .. .. .. .. .. .. .. .. .. | 1930 |
| **Klapp, R., and Block, W.** | *Die Knochenbruchbehandlung mit Drahtzugen* (6) | 1930 |
| **Lindsay, J. A.** .. .. | *Among the Thinkers* .. .. .. .. .. .. .. | 1931 |
| **Matti, H.** .. .. .. | *Die Knochbrüche. 2te Aufl.* .. .. .. (6) | 1931 |
| **Nesfield, V.** .. .. | *Deafness and its Alleviation* .. .. 2nd Ed. | 1931 |
| **Power, Sir D'Arcy** .. | *Medicine in the British Isles* .. .. .. | 1930 |
| **Rawling, L. B.** .. | *Stepping-Stones to Surgery* .. .. .. .. .. | 1930 |
| **Rolleston, Sir H.** .. | *Internal Medicine* .. .. .. .. .. .. .. .. | 1930 |
| **Pye, W.** .. .. .. .. | *Surgical Handicraft* .. .. .. .. .. .. .. .. | 1931 |
| **Spencer, W. G., and Cade, S.** | *Diseases of the Tongue* .. .. 3rd Ed. | 1931 |
| *System of Bacteriology* | .. .. .. .. .. .. .. Vols. 6 and 8 (8) | 1931 |
| **Thomson, Sir St. C., and Colledge, L.** | *Cancer of the Larynx* .. .. | 1930 |
| **Wilson, G. E.** .. .. | *Fractures and their Complications* .. (6) | 1931 |
| **Yeomans, P. W.** .. | *Happy Motherhood* .. .. .. .. .. .. .. .. | 1931 |

TRANSACTIONS, REPORTS, JOURNALS, ETC.

| | |
|---|---|
| American Laryngological Association, Transactions. Vol. 52 .. (2) | 1930 |
| American Laryngological, Rhinological and Otological Association, Transactions. Vol. 36 .. .. .. .. .. .. .. .. .. .. .. (3) | 1930 |
| Henry Phipps Institute, 22nd Annual Report .. .. .. .. .. (7) | 1930 |
| Index Catalogue of Surgeon-General's Library. 3rd Series. Vol. 9 (10) | 1931 |
| Medical Annual, 1931 .. .. .. .. .. .. .. .. .. .. .. .. .. | 1931 |
| Ophthalmological Society of the U.K., Transactions. Vol. 50 .. | 1930 |
| Progressive Medicine, March, 1931 .. .. .. .. .. .. .. .. .. .. | 1931 |
| Quarterly Cumulative Index Medicus. Vol. 8 .. .. .. .. .. .. | 1931 |
| Report on Sanitary Department, Gold Coast, 1929–30 .. .. .. .. | 1930 |
| Société Internationale de Chirurgie, 8me Congrés. Vol. 2 .. (6) | 1930 |
| Surgical Clinics of North America, February and April, 1931 .. | 1931 |
| University of Otago Medical School, Proceedings. No. 8 .. (9) | 1931 |

### Local Medical Notes

EXAMINATION RESULTS.

**University of Bristol.**—Students of the University have recently passed the following examinations :—

M.D.—S. Datta.

M.B., Ch.B. (*Part I.*).—*Second Examination. Pass :* V. T. Baxter. *In Elementary Anatomy only :* D. S. Prichard. *In Organic Chemistry only :* Ursula W. Wood.

M.B., Ch.B. (*Part II.*).—*Second Examination. Pass :* G. L. L. Gurney, T. R. V. Gurney, Irene G. Hamilton, F. J. W. Lewis, C. H. G. Price.

M.B., Ch.B. (*Part I.*). — *Final Examination. Pass :* H. M. F. Finzel (with distinction in Materia Medica, Pharmacy,

Pharmacology and Therapeutics, and Forensic Medicine and Toxicology), J. J. Kempton, R. L. Marks.

M.B., CH.B. (*Part II.*).—*Final Examination. Pass :* Elizabeth S. G. Owen (with Second Class Honours), L. P. Ashton (with distinction in Surgery and Obstetrics), J. E. Brown, L. R. Jordan (with distinction in Medicine), R. H. Moore. *In Group II.* (*Completing Examination*) *:* Eveline M. D. M. Collinson, Rowena M. Hickman. *In Group I. only :* W. A. F. Taylor. *In Group II. only :* O. J. P. Bollon.

B.D.S.—*Second Examination. Pass : In Anatomy and Physiology and Histology* (*Completing Examination*) *:* J. W. E. Snawdon.

B.D.S.—*Final Examination. Pass :* M. J. Ryan.

PRELIMINARY SCIENCE EXAMINATION FOR DENTAL STUDENTS.—*In Chemistry and Physics only. Pass :* R. E. Buchan, Frances M. Orton.

L.D.S.—*First Professional Examination. Pass : In Dental Anatomy* (*Completing Examination*) *:* J. H. G. Smith. *In Anatomy and Physiology only :* J. G. Clancy.

L.D.S. — *Second Professional Examination. Pass : In Dental Mechanics and Dental Metallurgy* (*Completing Examination*) *:* G. N. Minifie. *In Dental Mechanics and Dental Metallurgy only :* R. L. Royal. *In Dental Materia Medica only :* I. R. R. Eskell.

L.D.S.—*Final Professional Examination. Pass :* E. V. O'Hara.

**Conjoint Board**—M.R.C.S., L.R.C.P.—*In Chemistry and Physics :* J. G. Jones, J. H. Bulleid. *In Chemistry only :* Mabel L. Glenny. *In Anatomy and Physiology :* C. H. G. Price. *In Pharmacology and Materia Medica :* A. F. Fowler, D. P. Dewe, W. R. Cambridge. *In Medicine :* R. G. Wilbond,* R. H. Moore,* D. Johnson, H. L. Cleave,* Elizabeth S. G. Owen.* *In Surgery :* H. L. Cleave,* L. C. Holland, Yolande M. de la Pasture,* Elizabeth S. G. Owen,* Ida B. Saxby.* *In Midwifery :* D. Johnson, H. L. Cleave,* Yolande M. de la Pasture,* N. C. Coombs, F. W. A. Fosbery, R. V. C. Richards.*

L.D.S., R.C.S.—*First Professional Examination. Part I.* (*Dental Mechanics*) *:* D. Gent. *Part II.* (*Dental Metallurgy*) *:* D. Gent, J. D. Rees, H. F. Vere, B. H. W. Baker, A. C. Davies, L. Jonathan, N. E. Miles, G. L. R. Welch. *Part III* (*b*). (*Dental Anatomy and Physiology*) *:* S. A. McCandlish.

* Qualified.

### Publications Received

From B. FRASER & Co.

*Tuberculosis—its Treatment and Cure with the Help of Umckaloabo.* By an English Physician, M.R.C.S., L.R.C.P. Price 3s. 6d.

From WILLIAM HEINEMANN (MEDICAL BOOKS) LTD.:

*Restoration Exercises for Women.* By E. A. HORNIBROOK. Price 5s.
*Handbook of Diets.* By ROSE M. SIMMONDS, S.R.P. Price 7s. 6d.
*Happy Motherhood.* By PEGGY WINEFRED YEOMANS. Price 3s. 6d.

From THE LANCET LTD.:

*Guy's Hospital Reports.* January, 1931, and April, 1931. Price 12s. 6d. each part.

From H. K. LEWIS & CO. LTD.:

*The Alcohol Habit and its Treatment.* By W. E. MASTERS, M.D., F.R.C.S., D.P.H. Price 6s.
*The History of Medicine.* By B. DAWSON, M.D., F.R.C.S. Price 7s. 6d.
*Among the Thinkers.* By J. A. LINDSAY, M.A., M.D., F.R.C.P. Price 5s.
*Conduct of Life Assurance Examinations.* By E. M. BROCKBANK, M.D., F.R.C.P. Price 7s. 6d.
*Physical and Radiological Examination of the Lungs.* 2nd Edition. J. CROCKET, M.D., D.P.H., F.R.C.P.E. Price 16s.

From SAMPSON LOW, MARSTON & CO. LTD.:

*Sixty Years of Health and Physick.* By S. G. BLAXLAND STUBBS and E. W. BLIGH. Price 15s.

From NATIONAL TEMPERANCE LEAGUE:

*Medical Evidence Before the Royal Commission on Licensing,* 1930. By COURTENAY C. WEEKS, M.R.C.S., L.R.C.P. Price 1s.

From OXFORD UNIVERSITY PRESS:

*Injuries and Sport.* By C. B. HEALD, C.B.E., M.A., M.D., M.R.C.P. Price 25s.
*Primary Syphilis in the Female.* By THOMAS ANWYL DAVIES, M.D. Price 12s. 6d.

From THE UNIVERSITY OF WARSAW:

*Travaux Scientifiques de la Clinique Infantile de l'Université de Varsovie,* 1921–1929.

From JOHN WRIGHT & SONS LTD.:

*British Journal of Surgery.* Vol. xiii. No. 72. April, 1931. Price 12s. 6d.
*The Cardiac Cycle.* By HARRINGTON SAINSBURY, O.B.E., M.D., F.R.C.P. Price 5s.
*Medical Annual.* 49th Year. 1931. Price 20s.

